# “Quando o país depende inteiramente dos parceiros, as coisas tendem a não dar certo”: iniciativas globais de saúde e HIV/aids em Guiné-Bissau

**DOI:** 10.1590/0102-311XPT151324

**Published:** 2025-11-07

**Authors:** Amiry Monteiro Sanca, Franciela Delazeri Carlotto, Cristianne Maria Famer Rocha, Deise Lisboa Riquinho

**Affiliations:** 1 Escola de Enfermagem, Universidade Federal do Rio Grande do Sul, Porto Alegre, Brasil.

**Keywords:** HIV, Cooperação Internacional, Política de Saúde, Instituições de Caridade, HIV, International Cooperation, Health Policy, Charities, VIH, Cooperación Internacional, Política de Salud, Organizaciones de Beneficencia

## Abstract

O objetivo deste estudo foi compreender a participação das iniciativas globais de saúde (IGS) no enfrentamento da epidemia de HIV/aids em Guiné-Bissau, a partir de distintos atores sociais e políticos, representantes das IGSs, do governo guineense e das organizações da sociedade civil. Foi realizado um estudo de caso de abordagem qualitativa, do tipo descritivo exploratório, que utilizou entrevista semiestruturada com 36 participantes. Empregou-se técnica de análise de conteúdo temática. Os relatos dos participantes foram organizados e apresentados a partir de quatro categorias temáticas. Os resultados indicam que, na resposta ao HIV/aids, além do governo, identificou-se pelo menos 15 instituições não governamentais e uma forte parceria com o Brasil. As limitações e desafios do governo estão relacionados à captação de financiamento e gerenciamento dos recursos proveniente das IGSs. As contribuições das IGSs foram descritas como positivas, pois há um desfinanciamento interno e dependência externa onde o Fundo Global se confirma como o principal financiador das ações de resposta ao HIV/aids. Essa dependência interfere na autonomia da gestão e afeta a coordenação. A realidade da Guiné-Bissau se assemelha a vários países da África Subsaariana onde a atuação das IGSs é caracterizada por um paradoxo: a dependência dos recursos financeiros e a necessidade de se descolonizar da ajuda externa que, de certa forma, atua como um dispositivo disciplinador. Os achados evidenciam a necessidade do país de adotar as características de um Estado estável, seguro e com medidas que instigam sua autonomia e independência da colonialidade.

## Abstract (en)

The aim of the present study was to understand the participation of global health initiatives (GHIs) in addressing the HIV/AIDS epidemic in Guinea-Bissau based on the insights of different social and political actors, representatives of GHIs, the Guinean government, and civil society organizations. A qualitative, descriptive, exploratory case study was conducted involving interviews with 36 participants. Thematic content analysis was employed. The discourses of the participants were organized into four main topics. The results indicate that, besides the government, at least 15 nongovernmental organizations and a strong partnership with Brazil were identified in the response to HIV/AIDS. The limitations and challenges of the government are related to the acquisition of funding and the management of the resources provided by GHIs. The contributions of GHIs were described as positive, considering the internal lack of funding and external dependence, with the Global Fund as the primary funder of HIV/AIDS response actions. This dependence interferes with the autonomy of management and affects coordination. The situation in Guinea-Bissau resembles that of several countries in Sub-Saharan Africa, in which the work of GHIs is characterized by a paradox: dependence on financial resources and the need to decolonize from external aid, which, to some extent, serves as a disciplinary device. The findings underscore the need for the country to adopt characteristics of a stable government, with measures that ensure its autonomy and independence from colonialism.

## Abstract (es)

El objetivo de este estudio fue comprender la participación de las iniciativas globales de salud (IGS) en la lucha contra la epidemia de VIH/sida en Guinea-Bisáu desde diferentes actores sociales y políticos, representantes de IGSs, el Gobierno guineano y las organizaciones de la sociedad civil. Se realizó un estudio de caso con enfoque cualitativo, de naturaleza descriptiva exploratoria, a partir de una entrevista semiestructurada con 36 participantes. La técnica aplicada fue el análisis de contenido temático. Los relatos de los participantes se organizaron y se presentaron con base en cuatro categorías temáticas. Los resultados revelan que las acciones de respuesta al VIH/sida provinieron de, al menos, 15 instituciones no gubernamentales y del Gobierno, y que hay una fuerte colaboración con Brasil. Las limitaciones y desafíos del gobierno se relacionan con la recaudación de fondos y la gestión de los recursos de las IGS. Las contribuciones de las IGSs fueron positivas, pues existe una definición interna y una dependencia externa donde el Fondo Mundial se confirma como el principal financiador de las acciones de respuesta al VIH/sida. Esta dependencia interfiere con la autonomía de gestión y afecta a la coordinación. La realidad de Guinea-Bisáu se asemeja a la de varios países del África subsahariana donde el desempeño de las IGSs se caracteriza por una paradoja: la dependencia de los recursos financieros y la necesidad de descolonizar de la ayuda externa que, en cierto modo, actúa como un dispositivo disciplinario. Los hallazgos destacan que el país debería adoptar características de un Estado estable, seguro y con medidas que promuevan su autonomía e independencia de la colonialidad.

## Introdução

As iniciativas globais de saúde (IGS) são parcerias globais que foram formadas no início dos anos 2000, com vistas a contribuir no aperfeiçoamento dos sistemas e serviços de saúde dos países subdesenvolvidos e reverter o curso do HIV, da aids, da tuberculose e da malária, prioritariamente, com foco em intervenções que visam auxiliar os países subdesenvolvidos na concretização dos Objetivos do Desenvolvimento do Milênio (ODM). Essas iniciativas são caracterizadas por lidar com questões de interesse internacional e executar programas de grande alcance e de financiamento em vários países, por meio de apoio envolvendo novos e velhos parceiros, tanto do setor público como do privado ^1^ ^,^ ^2^.

O Plano de Emergência do Presidente dos Estados Unidos para Alívio da Aids (PEPFAR, acrônimo em inglês), o Fundo Global de Combate à Aids, Tuberculose e Malária (Fundo Global) e o Programa Multipaíses de Aids do Banco Mundial configuram as três maiores IGSs, respondendo por dois terços do financiamento externo para HIV ^1^. *Pari passu*, o continente africano, principalmente a região Subsaariana, tem se destacado como área mais atingida pelo HIV desde o seu surgimento, expressando maiores índices de morbimortalidade por causas relacionadas à aids e concentrando aproximadamente dois terços de todas as pessoas vivendo com HIV no mundo ^3^. Nesta ótica, em decorrência da fragilidade dos sistemas de saúde dos países subsaarianos e das altas taxas de morbimortalidade por doenças infecciosas como o HIV, muitas IGSs atuam nesta região ^4^.

De igual modo aos países subsaarianos, na Guiné-Bissau, país africano de língua oficial portuguesa, o HIV/aids constitui um problema à saúde pública e oferece desafios para os gestores e profissionais. A prevalência do HIV em adultos (de 15 a 49 anos) nesse país foi de 2,4% no ano de 2022 e, com exceção de Guiné Equatorial (taxa de prevalência de 6,7%) e Moçambique (com indicador indisponível), a Guiné-Bissau tem a maior taxa entre os Países Africanos de Língua Oficial Portuguesa (PALOP), sendo que Angola possui uma taxa de 1,5%, São Tomé e Príncipe uma taxa de 0,4% e Cabo Verde uma taxa de 0,9%. O mesmo ocorre em comparação aos países com os quais Guiné-Bissau faz fronteiras: Guiné (taxa de 1,4%) e Senegal (taxa de 0,3%), todos situados na África Subsaariana. Guiné-Bissau apresenta, ainda, maior prevalência em comparação com outros países de língua oficial portuguesa que não pertencem à África: Brasil (taxa de 0,6%), Portugal (taxa de 0,5%) e Timor-Leste (taxa de 0,2%) ^5^.

Nesta conjuntura, considerando a finalidade das IGSs, o desafio do enfrentamento à epidemia de HIV/aids em Guiné-Bissau e os esforços para a erradicação da aids até 2030, conhecer e identificar a atuação das IGSs presentes no país e suas participações pode servir de base para que atores sociais, pesquisadores e formuladores de políticas nacionais e internacionais explorem diferentes aspectos dessa participação em relação ao HIV/aids no país, assim como estimular reflexões. Vale ressaltar que, até onde se tem conhecimento (buscas realizadas em bases de dados, como MEDLINE/PubMed, Embase, Web of Science, Scopus e Google Acadêmico, nos idiomas inglês, português, espanhol e francês e sem limite de ano) ^4^, não há artigos científicos que analisam a participação das IGSs no enfrentamento da epidemia de HIV/aids em Guiné-Bissau, a partir de distintos atores sociais e políticos.

A partir dessas intenções mais gerais, questiona-se: como ocorre a participação das IGSs no enfrentamento da epidemia de HIV/aids em Guiné-Bissau? Assim, este estudo teve como objetivo compreender a participação das IGSs no enfrentamento do HIV/aids em Guiné-Bissau, a partir de distintos atores sociais e políticos, representantes das IGSs, do governo guineense e das organizações da sociedade civil (OSC).

## Método

Estudo de caso de abordagem qualitativa, do tipo descritivo exploratório. Concebido como uma investigação empírica que se debruça sobre um fenômeno contemporâneo (o caso) em profundidade e em seu próprio contexto de mundo real, principalmente quando não há limites claramente evidentes entre o fenômeno e o contexto ^6^. Para Minayo ^7^, esta modalidade de estudo utiliza estratégias de pesquisa para mapear, descrever e analisar o contexto, as relações e as percepções sobre determinada situação ou fenômeno.

O estudo ocorreu na República da Guiné-Bissau e contou com 36 participantes, sendo 17 membros da Rede Nacional de Associações de Pessoas com HIV (RENAP), 13 gestores do Programa Nacional de Luta contra Sida e Hepatites Virais (PNLS/HV) e/ou coordenadores dos Centros de Tratamento Ambulatorial (CTA) e 6 representantes das principais IGS e instituições não governamentais (ONG) que atuam na resposta nacional ao HIV/aids. Todos os entrevistados estavam ativos em suas funções e foram selecionados, de forma intencional, por meio de convite pessoal em abordagem presencial. Adotaram-se como critérios de inclusão ter o domínio da língua portuguesa ou crioula, ter idade igual ou superior a 18 anos, pertencer a um dos grupos acima referidos e estar exercendo o cargo/função há mais de um ano ou ter desempenhado cargo/função nos últimos cinco anos e permanecido nele ao menos por dois anos. A apresentação de algum comprometimento de comunicação (pessoa com deficiência auditiva ou perda da capacidade de fala) ou afastamento do trabalho (por férias, adoecimento, viagem de trabalho etc.) durante o período de coleta das informações foi usada como critério de exclusão. Dentre as pessoas convidadas, três não participaram da pesquisa alegando falta de tempo.

As informações foram coletadas por um pesquisador nativo, enfermeiro, nos espaços de preferência dos participantes, por meio de entrevista semiestruturada, no período de dezembro de 2023 a fevereiro de 2024. Todas as entrevistas foram gravadas, com duração média de 50 minutos e, posteriormente, transcritas na íntegra. O roteiro incluiu aspectos como a atuação das IGSs no enfrentamento ao HIV na Guiné-Bissau (identificação/mapeamento, atividades e efeitos das entidades internacionais no enfrentamento ao HIV), atuação do governo no enfrentamento ao HIV, papel dos “ativistas” na resposta ao HIV e limitações/desafios no combate ao HIV na Guiné-Bissau. 

O estudo não buscou representatividade numérica, mas sim um aprofundamento da temática pesquisada. Portanto, o tamanho da amostra foi orientado pelo princípio do “poder da informação” que, segundo Malterud et al. ^8^, determina que o tamanho de uma amostra com poder de informação suficiente depende do objetivo do estudo, da especificidade da amostra, do uso da teoria estabelecida, da qualidade do diálogo e da estratégia de análise.

A análise temática de conteúdo foi a técnica implementada para analisar as informações, desenvolvendo-se em três fases: pré-análise; exploração do material; e tratamento dos resultados obtidos/interpretação ^9^. As informações foram organizadas e apresentadas a partir de quatro categorias temáticas: (1) Combate à epidemia do HIV: presença e atuação das IGSs e ONGs; (2) Limitações e desafios do governo na coordenação dos recursos; (3) Financiamento do combate à epidemia do HIV; e (4) Utilização dos recursos das IGSs.

A pesquisa foi aprovada em Guiné-Bissau pelo Comitê Nacional de Ética na Saúde (referência nº 035 CNEPS/INASA/2023) e teve anuência do Ministério da Saúde do país (referência nº 101/DGPPS/2023). Com o intuito de preservar a identidade dos participantes, foram utilizados nomes fictícios e indicados os segmentos aos quais pertencem (I para IGS e ONG, G para governo e R para membros da RENAP) na descrição dos resultados. O desenvolvimento do estudo seguiu os critérios do *Consolidated Criteria for Reporting Qualitative Research* (COREQ; Critérios Consolidados para Relatórios de Pesquisa Aualitativa) ^10^.

Foram identificadas 15 IGSs e ONGs de combate à epidemia de HIV/aids na Guiné-Bissau, por meio dos depoimentos dos participantes, conforme apresentado no Quadro 1.

**Quadro 1:** Principais iniciativas globais de saúde (IGS) e organizações não governamentais (ONG) de combate à epidemia do HIV em Guiné-Bissau.

NOME	DESCRIÇÃO	LINK *
Fundo Global de Combate à Aids, Tuberculose e Malária (Fundo Global)	Organização financeira internacional (parcerias público-privadas) criada em 2002, que angaria e distribui fundos para tratamento de aids, tuberculose e malária. Atua em Guiné-Bissau desde 2004	https://anaisihmt.com/index.php/ihmt/article/view/228/189
Organização Mundial da Saúde (OMS)	O acordo de base foi rubricado em 14 de setembro de 1974, mas só em 1976 se abriu uma representação no país	https://www.afro.who.int/sites/default/files/2017-06/ccs_guineebissau_2009_2013_po.pdf
Programa Conjunto das Nações Unidas sobre HIV/Aids (UNAIDS)	Criado em 1996, o UNAIDS tem liderado o esforço global para acabar com a aids, enquanto uma ameaça à saúde pública até 2030, como parte dos Objetivos de Desenvolvimento Sustentável (ODS)	https://www.unaids.org/en/whoweare/about
Fundo das Nações Unidas para a Infância (UNICEF)	Criado em 1946, trabalha em mais de 190 países e territórios para proteger os direitos de todas as crianças	https://www.unicef.org/
Fundo de População das Nações Unidas (FNUAP)	É a agência de saúde sexual e reprodutiva da Organização das Nações Unidas (ONU), trabalha para acelerar o acesso universal à saúde sexual e reprodutiva, incluindo o planejamento familiar voluntário e a maternidade segura; busca a efetivação dos direitos e oportunidades para as pessoas jovens e auxilia na aquisição de produtos essenciais para prevenir e tratar infecções sexualmente transmissíveis	https://guinea-bissau.unfpa.org/pt
Programa Alimentar Mundial (PAM)	Desenvolve apoio à saúde e nutrição, alimentação escolar e desenvolvimento rural. Presta assistência técnica ao governo para ajudar o país a reduzir a subnutrição, aumentar o acesso à educação e melhorar o controle da segurança alimentar	https://www.impactpool.org/jobs/932633
ENDA Santé	Organização internacional sem fins lucrativos, com sede no Senegal. Responsável pelos programas de saúde no âmbito da ENDA Tiers Monde, juntamente com outras 24 áreas temáticas. Desde 1988, tem colaborado com organizações comunitárias de base, estruturas governamentais e internacionais na implementação de ações programáticas para combater a aids, a malária e promover iniciativas comunitárias de saúde no Senegal e na África Ocidental	https://www.enda-sante.org/notre-histoire/
PLAN Internacional	ONG internacional, iniciou as atividades em 1995, trabalha para garantir que todas as crianças vivam em uma sociedade onde seus direitos à educação, saúde e direitos sexuais e reprodutivos sejam garantidos, inclusive em situações de emergência	https://nanomon.org/redes/plan-internacional-guine-bissau; https://plan-international.org/guine-bissau/
Ajuda de Desenvolvimento de Povo para Povo - Guiné-Bissau (ADPP-GB)	A ADPP-GB é uma organização não governamental sediada na Guiné-Bissau e um membro da Federação de Associações Ligadas ao Movimento Internacional Humana People to People. Sua missão é apoiar a população em geral e as comunidades mais necessitadas por meio de projetos de desenvolvimento econômico, social e cultural, com o propósito de aumentar o bem-estar das pessoas e encorajar a sua participação ativa	https://www.adpp-gb.org/quem-somos#sobre-nos
Centro de Informação Despistagem e Aconselhamento da ONG Alternag (CIDA Alternag)	Oferece serviços de enfermagem e médico, especialmente nas áreas de testagem voluntária e aconselhamento do HIV/aids. Atua em várias regiões do país (Bissau, Bafatá, Gabú, Oio, Quinara, Tombali, Cacheu e Biombo), trabalhando preferencialmente com mulheres grávidas	https://www.convencaocidada.gw/assets/tematicas/anexos/Relato%CC%81rio%20-%20Sau%CC%81de.pdf; https://saude.cplp.org/media/vz3abzdo/cplp_guine-bissau_2018.pdf
Associação Céu e Terra	Fundada em 2001, por iniciativa de uma médica cubana, outrora residente no país, e um padre da igreja católica. A associação promove assistência clínica às pessoas vivendo com HIV/aids e capacitação do pessoal técnico para sensibilização e prevenção da infecção do HIV/aids. Pioneira e referência na prevenção da transmissão vertical do HIV no país	https://www.teses.usp.br/teses/disponiveis/6/6136/tde-21022014-085211/publico/SuadoSane.pdf
Associação Guineense para o Bem Estar Familiar (AGUIBEF)	Fundada em 1987, organização sem fins lucrativos não governamental, especializada na saúde sexual e reprodutiva. É membro associado da Federação Internacional para o Planejamento Familiar (IPPF)	https://aguibef.org/home/
Agência Guineense Marketing Social (AGMS)	Oferece serviços de prevenção de HIV/aids e desenvolve atividades de promoção do uso de preservativos em todo o país, atuando preferencialmente em Bissau e em regiões como Oio, Tombali, Quinara e São Domingos	https://www.convencaocidada.gw/assets/tematicas/anexos/Relato%CC%81rio%20-%20Sau%CC%81de.pdf; https://saude.cplp.org/media/vz3abzdo/cplp_guine-bissau_2018.pdf
Rede Nacional de Associações de Pessoas com HIV (RENAP)	É uma rede de 15 associações de pessoas vivendo com o HIV, das quais sete atuam na cidade de Bissau. A sua força de intervenção comunitária assenta nos seus ativistas, os quais levam a cabo um conjunto de programas, visando uma maior implicação comunitária de pessoas vivendo com o HIV no combate à transmissão da infeção nos grupos mais vulneráveis, como as crianças, assegurando retorno dos doentes ao tratamento	https://repositorio.iscte-iul.pt/bitstream/10071/17846/1/master_sonia_silva_polonio.pdf

Fonte: elaboração própria a partir de informações publicadas nos links indicados.

* Acessados em 30/Jul/2024.

As IGSs alicerçam-se em quadros teóricos que articulam justiça social, governança global e determinantes estruturais da saúde. As abordagens críticas da governança global, como delineado pela Comissão Lancet/Universidade de Oslo ^11^, enfatizam que os determinantes políticos da saúde - incluindo políticas comerciais, financeiras e ambientais - operam além do setor sanitário, exigindo uma governança intersetorial que transcende as fronteiras dos países, bem como das instituições. Essas perspectivas defendem a formação de parcerias multilaterais híbridas, integrando atores estatais, organizações internacionais, setor privado e sociedade civil, a fim de promover transparência decisória, *accountability* distribuída e mecanismos de monitoramento extrassetorial ^11^. Tais elementos são fundamentais para enfrentar assimetrias de poder e interesses concorrentes na arena global. Sob esse prisma, as IGSs são concebidas não apenas como respostas técnicas a problemas biomédicos, mas como estratégias sistêmicas e ético-políticas, alicerçadas em estruturas jurídicas internacionais, financiamento equitativo e cooperação multilateral, com o objetivo de reduzir iniquidades sanitárias estruturais e afirmar a saúde como um direito humano universal.

## Resultados e discussão

A primeira categoria reúne informações/relatos sobre as principais IGSs e ONGs presentes na Guiné-Bissau, assim como as atuações no enfrentamento do HIV/aids, conforme consta no Quadro 2.

**Quadro 2:** Combate à epidemia do HIV em Guiné-Bissau: presença e atuação das iniciativas globais de saúde (IGS) e organizações não governamentais (ONG).

RELATOS DOS PARTICIPANTES
“*Os principais são: Fundo Global, OMS, UNAIDS, UNICEF, ENDA Santé, CIDA Alternag, Associação Céu e Terras, ADPP-GB e RENAP*” (Vitor G)
“*A ONU tem uma equipe conjunta para a resposta ao HIV na Guiné-Bissau. A equipe é composta por membros da FNUAP, UNICEF, PAM e OMS, tudo sob a coordenação do UNAIDS.* (...) *O UNAIDS não tem um escritório próprio no país, tem um ponto focal que atua dentro do escritório da FNUAP.* (...) *Cada uma das agências da ONU que compõem a equipe conjunta se responsabiliza por um eixo de intervenção de acordo com a divisão de tarefas previamente definidas. Por exemplo, a FNUAP atua com foco nas populações-chave para HIV, serviço integral, jovens, transmissão primária de HIV nas mulheres em idade fértil e prevenção de gravidez não desejada/planejada nas mulheres que vivem com HIV. A UNICEF na prevenção da transmissão vertical do HIV, diagnóstico infantil precoce e entre outras. A OMS atua mais na gestão de informações estratégicas, gestão de dados e apoio aos estudos sobre HIV e PVHIV. O PAM fornece apoio nutricional às PVHIV. A grosso modo, funciona dessa forma.* (...) *A equipe conjunta reúne-se regularmente para definir estratégias e presta suporte ao governo para definição das prioridades de intervenção que não foram financiadas pelo Fundo Global, assim como, também apoia as entidades não governamentais que atuam no enfrentamento do HIV. O novo Plano Estratégico da eliminação tripla: HIV, sífilis e hepatite B, conta com o cofinanciamento de UNICEF e FNUAP. A elaboração do novo Plano Estratégico de luta contra a aids, que vai de 2024 a 2028, contou com apoio técnico e financeiro do UNAIDS e elaboração da proposta de financiamento enviada para o Fundo Global*” (Zoe I)
“*Dos países, destaca-se a parceria com o Brasil. Temos cooperação na área técnica, formações e treinamentos dos profissionais, fornecimento de medicamentos, especialmente ARV e materiais de testagem. Alguns colegas de trabalho iam para treinamento na FIOCRUZ e em outras instituições brasileiras e alguns brasileiros vinham ministrar treinamentos aqui. O apoio do Brasil reduziu muito com a chegada do Fundo Global.* (...) *Temos uma sólida relação de intercâmbio de conhecimento com Senegal.* (...) *Eu acho que a Guiné-Bissau está dando pouca importância à cooperação com outros países em detrimento do Fundo Global. Estão focando mais em receber dinheiro do que o conhecimento técnico como o que o Brasil fornecia*” (Sofia G)
“*Em 2005, uma equipe de nove profissionais foi enviada ao FIOCRUZ, Brasil, para formação técnica. Ao regressar, produziram o protocolo de cuidado clínico às PVHIV. De 2004 a 2005, o Brasil doou os ARV para nosso país.* (...) *O Brasil ainda envia algumas doações de medicamentos para tratamento de hepatite B, preservativos e autotestes de HIV.* (...) *Das ONGs, tem ENDA Santé que foca nas populações-chave, a ADPP-GB também atua com população-chave e em muitas outras áreas tem apoiado as Forças Armadas no combate ao HIV. A PLAN Internacional também é um dos nossos colaboradores. AGMS na prevenção ao HIV, AGUIBEF no HIV, saúde sexual e reprodutiva testagem e tratamento de HIV, Associação Céus e Terras pioneiro e referência na prevenção de transmissão vertical de HIV e CIDA Alternag, também pioneira no rastreio voluntário de HIV*” (Mila G)
“*Dentre os pacotes de HIV no projeto financiado pelo Fundo Global, a PLAN Internacional ficou com o de prevenção difusão de conhecimento nas estruturas sanitárias e comunidades sobre a prevenção de HIV, acompanhamento domiciliar, buscas ativas das pessoas em abandono de ARV. Para tanto, trabalha em parceria com a RENAP* (...)*, a PLAN Internacional e a ADPP-GB têm os mesmo pacotes de intervenção no âmbito da subvenção do Fundo Global, porém, atuam em regiões diferentes...*” (Oliver I)

ADPP-GB: Ajuda de Desenvolvimento de Povo para Povo - Guiné-Bissau; AGMS: Agência Guineense Marketing Social; AGUIBEF: Associação Guineense para o Bem Estar Familiar; ARV: antirretrovirais; CIDA Alternag: Centro de Informação Despistagem e Aconselhamento da ONG Alternag; FIOCRUZ: Fundação Oswaldo Cruz; FNUAP: Fundo de População das Nações Unidas; Fundo Global: Fundo Global de Combate à Aids, Tuberculose e Malária; OMS: Organização Mundial da Saúde; ONU: Organização das Nações Unidas; PAM: Programa Alimentar Mundial; PVHIV: pessoas vivendo com HIV; RENAP: Rede Nacional de Associações de Pessoas com HIV; UNAIDS: Programa Conjunto das Nações Unidas sobre HIV/AIDS; UNICEF: Fundo das Nações Unidas para a Infância.

Os relatos dos participantes convergem reconhecendo a forte presença de organizações internacionais (IGS e ONG) no combate ao HIV na Guiné-Bissau, bem como a contribuição histórica do Brasil na resposta ao HIV (via Fundação Oswaldo Cruz [FIOCRUZ], fornecimento de antirretrovirais [ARV] e capacitação técnica), o Fundo Global como principal financiador das ações de combate ao HIV, Organização das Nações Unidas (ONU) e suas agências (Programa Conjunto das Nações Unidas sobre HIV/AIDS [UNAIDS], Organização Mundial da Saúde [OMS], Fundo das Nações Unidas para a Infância [UNICEF], Programa Alimentar Mundial [PAM], Fundo de População das Nações Unidas [FNUAP]) como estruturadas em um modelo de atuação conjunta, com eixos de intervenção bem definidos. ONGs como ENDA Santé, Ajuda de Desenvolvimento de Povo para Povo - Guiné-Bissau (ADPP-GB), PLAN Internacional, RENAP, Agência Guineense Marketing Social (AGMS), Associação Guineense para o Bem Estar Familiar (AGUIBEF), Associação Céus e Terras, Centro de Informação Despistagem e Aconselhamento da ONG Alternag (CIDA Alternag) são mencionadas por diversos participantes, com papéis consistentes: populações-chave, prevenção de transmissão vertical, testagem e tratamento, apoio às Forças Armadas e rastreio voluntário.

Alguns relatos evidenciam divergências na percepção sobre o impacto atual do Fundo Global e avaliação da cooperação internacional atual. Parte das falas sugere que houve uma perda de foco na autonomia técnica e no fortalecimento de capacidades locais, enquanto outras falas retratam o modelo atual como eficaz e estruturado.

Os resultados do estudo apontaram para uma presença massiva das IGSs e ONGs na resposta nacional ao HIV/aids, bem como a cooperação com o Brasil no domínio do enfrentamento ao HIV/aids. A cooperação entre os dois países é fruto do Programa Laços Sul-Sul (Cooperação Sul-Sul), envolvendo a doação de medicamentos ARV de primeira linha produzidos no Brasil, bem como a cooperação técnica em quaisquer das áreas temáticas do Centro Internacional de Cooperação Técnica em HIV/AIDS (CICT) ^12^. Como parte de tal cooperação, no primeiro semestre de 2024, o Brasil recebeu delegação de Guiné-Bissau para fortalecer a resposta ao HIV/aids, com ênfase em: prevenção comunitária ao HIV/aids e à tuberculose; prevenção da transmissão vertical de HIV, sífilis e hepatites virais; gestão e fortalecimento das áreas transversais para resposta ao HIV e à aids; e fortalecimento técnico na resposta ao HIV. No ano anterior, uma missão de prospecção do governo brasileiro foi à Guiné-Bissau, com o objetivo de analisar o cenário para repactuar o projeto Fortalecimento do Combate ao HIV e à Aids na Guiné-Bissau - Fase II ^13^.

Em relação às OSCs na Guiné-Bissau, foram oficialmente reconhecidas por lei em 1992 e atuam sobretudo nas áreas de saúde, atividades produtivas, educação, proteção de grupos vulneráveis e advocacy. Existem aproximadamente 96 OSCs atuando no país, destas, 51 são ONGs, financiadas geralmente por doadores externos. No entanto, há uma crítica da associação dessas organizações aos doadores por, muitas vezes, se sentirem obrigados a priorizar os interesses dos doadores em detrimento dos seus, a fim de conseguirem os financiamentos ^14^.

No campo da saúde, o trabalho realizado por essas organizações é considerado essencial para a resposta nacional à epidemia de HIV/aids na Guiné-Bissau. Para tanto, o governo conta com vários parceiros, dentre elas a CIDA Alternag, ENDA Santé, Associação Céu e Terra, AGMS, AGUIBEF e RENAP para implementação de ações estratégicas ^15^. O UNAIDS destacou que OSCs “*que vivem com, estão em risco de ou são afetadas pelo HIV são a linha de frente do progresso na resposta ao HIV*” ^16^. Porém, por vários fatores, essas organizações vêm enfrentando dificuldades/impedimentos para exercer sua liderança, seja por obstáculos regulatórios e políticos, limitações de capacidade e repressões à sociedade civil, limitações dos direitos humanos de comunidades marginalizadas ou a falta de financiamento. Tais dificuldades/impedimentos obstruem o progresso dos serviços de prevenção e tratamento do HIV/aids ^16^.

A segunda categoria, Limitações e desafios do governo na coordenação dos recursos, engloba as narrativas sobre as limitações e desafios do governo, em especial, do PNLS/HV na coordenação dos recursos financeiros para o enfrentamento do HIV/aids, como mostram os relatos do Quadro 3.

**Quadro 3:** Limitações e desafios do governo de Guiné-Bissau na coordenação dos recursos.

RELATOS DOS PARTICIPANTES
“*Há uma enorme dificuldade para conseguir os financiamentos total para os cinco eixos temáticos de atuação (combate ao HIV). Apenas tratamento e rastreio estão financiados pelo Fundo Global*” (Bia G)
“*...Quando adotamos a estratégia de testar e tratar, os recursos do Fundo Global foram direcionados massivamente para testagem e tratamento, deixando poucos recursos, fragilizando o eixo de prevenção*” (Mila G)
“*A Célula de Gestão é um departamento resultante de um acordo entre o governo e o Fundo Global.* (...) *São funcionários públicos e, ao mesmo tempo, contratados e pagos pelo Fundo. Há uma diferença salarial abismal com os demais funcionários do Ministério* [da Saúde Pública]*. O salário deles é muito, mas muito mais alto. Temos a mesma carga horária e desempenhamos quase a mesma função. É desmotivador para nós, profissionais que trabalham diariamente ao lado deles. Os profissionais que atuam na assistência recebem muito mal e sabem que as pessoas da Célula de Gestão recebem uma fortuna. Acho que estão desmotivados por serem mal remunerados. Isso impacta diretamente na resposta ao HIV*” (Sofia G; Artur G)
“*O Fundo Global canalizava seus recursos diretamente para o governo, mas houve um atrito entre as duas partes e o Fundo Global criou uma Célula de Gestão dentro do próprio Ministério da Saúde Púlibca para controlar os recursos investido. O dinheiro é depositado na conta do Ministério, gerenciado por esta Célula, tudo com aval do ministro de saúde* (...) *Geralmente, as atividades ficam acumuladas para o mês de novembro e dezembro, elas vêm se arrastando durante o ano todo por causa da burocracia*” (Clara G)
“*Chegando perto do final do prazo das intervenções e do limite para uso dos recursos solicitados, resta muito dinheiro e o governo precisa gastar porque solicitou aquele valor de orçamento. Geralmente o resto do recurso volta para o Fundo Global porque não foi usado*” (Aya G)
“*Mesmo que tenha sobrado dinheiro de uma atividade aprovada pelo Fundo Global, não se pode usar desse recurso para realizar outra atividade sem aprovação prévia do Fundo. Se usar sem aprovação, o governo é obrigado a devolver o dinheiro. Caso contrário, ocorre o bloqueio de todos os recursos até a devolução do recurso de uso indevido. O peso dessas ações acaba recaindo sobre o gestor que aprovou o uso*” (Elias G)
“*Desde os recursos do Banco Mundial até o de Fundo Global, percebe-se que os gestores estão mais preocupados e focados em coordenar os recursos financeiros (captar, gerenciar e prestar conta) do que coordenar realmente as ações nacionais de luta contra o HIV. Focam-se mais na produção dos relatórios, justificativas de uso dos recursos e não sobra tempo para fazer o mais importante: a coordenação das atividades*” (Mila G)
“*Tanto os pontos focais nos ministérios, como as Células Regionais para respostas ao HIV, desapareceram por falta de recursos/financiamentos. Existiram quando o Banco Mundial financiava a luta contra o HIV, até 2009, mas quando o Fundo Global assumiu o financiamento, foram extintos por falta de recursos.* (...) *Os recursos do Banco Mundial eram empréstimos para o país, o qual tinha liberdade de usar como bem entendesse, enquanto que os de Fundo Global são doações, com menos liberdade para o uso. Previamente ao recebimento das doações, são definidos onde o governo pode ou não aplicar tais recursos. Porque é um acordo assinado com o Fundo Global. O recurso financiado não cobre as necessidades, então o país define onde tais recursos devem ser aplicados antes de recebê-los. Mudar depois o foco de aplicação do recurso que é difícil. Tem se priorizado a compra dos medicamentos, o que consome mais de 30% do recurso*” (Bia G)
“*Pela necessidade de apoio, o governo aceita com muita facilidade assinar parcerias, mas no fim, não monitora as atividades desses parceiros*” (Sofia G)
“*O PNLS/HV estava sem coordenador efetivo há dois anos.* (...) [Seria necessário] *revisar linha por linha todos os acordos com os parceiros nacionais e internacionais de modo a assumir o real protagonismo e fiscalizar o cumprimento dos acordos. Há uma grande fragilidade institucional na resposta ao HIV.* (...) *É preciso uma liderança institucional mais forte por parte do Ministério da Saúde Pública para que os recursos das IGSs sejam melhor utilizados. Em comparação com outros países africanos, os nossos recursos destinados ao combate do HIV são migalhas. Além do mais, não são bem aplicadas. No momento, o país não consegue utilizar todos os recursos para o enfrentamento do HIV. De modo geral, todos os programas do Ministério devolvem dinheiro para os financiadores no final do ano. É algo recorrente. Porém, muitas atividades ficam sem ser realizadas. Há programas que chegam a utilizar menos de 50% dos recursos destinados. Há muita dificuldade de executar o cronograma das atividades propostas anualmente. Pela primeira vez, o PNLS/HV está tendo um plano anual de atividades. O que tem acontecido, ao longo dos anos, são execuções dos planos anuais de atividades definidos com cada parceiro, a exemplo, do plano anual das atividades da UNICEF e do Fundo Global* (...)*. A subutilização dos recursos disponibilizados está associada principalmente com a falta de programação interna. Há muita burocracia para liberação dos fundos destinados ao combate do HIV, o que demanda tempo para a tramitação dos documentos. Então, é necessário que os recursos sejam solicitados com antecedência para que dê tempo de ser processado e liberado dentro do prazo. Não se pode deixar para solicitar quando faltam apenas alguns dias para realização da atividade proposta. Há necessidade de criação de um pequeno protocolo interno para facilitar*” (Arthur G)

Fundo Global: Fundo Global de Combate à Aids, Tuberculose e Malária; PNLS/HV: Programa Nacional de Luta contra Sida e Hepatites Virais; UNICEF: Fundo das Nações Unidas para a Infância.

Os participantes da pesquisa apontam, de forma convergente, diversas fragilidades na resposta ao HIV, com destaque para o financiamento limitado e excessivamente direcionado do Fundo Global, que prioriza ações como tratamento e rastreio, deixando a prevenção em segundo plano. Há consenso quanto à excessiva padronização desses recursos, cuja realocação exige autorização externa, dificultando a adaptação às necessidades locais. Também são mencionados os desafios com a burocracia que atrasa a execução das atividades e leva à devolução de recursos não utilizados. A falta de coordenação estratégica é outro ponto crítico, com gestores focados na prestação de contas, ausência de monitoramento das parcerias e vacância prolongada na liderança do PNLS/HV. Por fim, há críticas à dependência de financiadores externos, sobretudo ao modelo inflexível do Fundo Global, considerado mais exigente que o Banco Mundial, contribuindo para uma aplicação ineficaz dos recursos, inferior à observada em outros países africanos.

No entanto, os relatos também revelaram divergências entre os participantes da pesquisa em relação a vários aspectos da gestão e execução das ações. Quanto à Célula de Gestão, há uma crítica mais contundente de alguns participantes, que apontaram seu impacto negativo sobre a motivação da equipe do Ministério da Saúde Pública, especialmente pelos entraves operacionais e burocráticos, enquanto outros não enfatizaram esse aspecto. Em relação à subutilização dos recursos, parte dos participantes atribui o problema à falta de programação interna e à carência de protocolos simples, defendendo que o foco excessivo em gestão financeira e relatórios compromete a coordenação das ações; por outro lado, há quem destaque as restrições externas impostas pelo Fundo Global como principal obstáculo. Da mesma forma, na avaliação da estratégia de financiamento, alguns veem as limitações do Fundo Global como entrave estrutural à flexibilidade da resposta nacional, enquanto outros atribuem maior peso a falhas internas de gestão e liderança. Por fim, apenas uma parte dos participantes menciona como crítica a falta de monitoramento das atividades dos parceiros pelo governo, o que não aparece com a mesma ênfase nas demais falas.

Estudos ^17^ ^,^ ^18^ ^,^ ^19^ realizados em Tanzânia, Burundi e Nigéria destacaram a existência de falta de liderança, fraca coordenação política e falta de visão política dos gestores de saúde. Na Zâmbia, no Malawi e no Quênia ^20^, foi constatada forte preocupação dos doadores internacionais em relação à capacidade administrativa dos governos de gerenciar de forma eficiente os volumosos recursos financeiros. No presente estudo, fatores como a alta dependência do financiamento externo, constante risco de bloqueio dos recursos financeiros oriundos das IGS e pseudo-autonomia na gestão e no uso desse recursos podem instigar a priorização da coordenação dos recursos financeiros (captar, gerenciar e prestar conta), em detrimento da coordenação das ações nacionais de resposta ao HIV/aids, ampliando a fragilidade na coordenação das políticas nacionais.

A terceira categoria, Financiamento do combate à epidemia do HIV, é composta por três subcategorias: (i) Avaliação das contribuições/financiamento das IGSs; (ii) Desfinanciamento interno e dependência externa; e (iii) Impacto do financiamento no planejamento e sustentabilidade das ações a longo prazo. Nesta categoria, foram incluídos relatos sobre a avaliação dos participantes em relação às contribuições/financiamento das IGSs no combate ao HIV/aids, a (não) participação do governo no financiamento das atividades de enfrentamento ao HIV/aids e o impacto da atual situação do financiamento no planejamento e sustentabilidade das ações a longo prazo, conforme ilustram os relatos do Quadro 4.

**Quadro 4:** Financiamento do combate à epidemia do HIV em Guiné-Bissau.

AVALIAÇÃO DAS CONTRIBUIÇÕES DO FINANCIAMENTO DAS IGSs
“*Se o Fundo Global retirar a sua ajuda à Guiné-Bissau, todos nós iremos nos infectar e morrer de aids. Não temos recursos para comprar ARV, testes e outros insumos para diagnóstico, prevenção e tratamento do HIV*” (Lara R)
“*Avalio de positiva e indispensável, pois sabemos que o nosso governo não tem capacidade de assegurar plenamente a execução das atividades de enfrentamento ao HIV.* (...) *Temos necessidade de pesquisas para subsidiar a nossa tomada de decisão, mas estamos com dificuldades de realizá-las. Toda ajuda é bem-vinda*” (Clara G)
“*O Fundo Global é quem financia mais de 90% das ações de luta contra o HIV. O Brasil também tem apoiado bastante, bem como outros parceiros internacionais*” (Zoe I)
“*Importante e crucial para as nossas atividades de combate ao HIV. O Fundo Global financia mais de 95% dos gastos com o combate ao HIV no país.* (...) *A saúde, de modo geral, é sustentada pelas IGSs. O enfrentamento das três principais doenças do país, HIV, TB e paludismo, é sustentado pelo Fundo Global*” (Bia G)
“*Os efeitos das entidades internacionais no enfrentamento ao HIV são positivos, porém, o financiamento é insuficiente.* (...) *Se não fosse pela ajuda dessas iniciativas estaríamos vivenciando uma catástrofe*” (Vitor G)
“*Vou focar no Fundo Global,* [que] *nos ajuda de todas as formas. Não recebemos o financiamento que costumamos solicitar, no entanto, sabemos que não somos os únicos necessitados e os recursos são escassos para financiar tudo o que os países pedem*” (Rosa G)
DESFINANCIAMENTO INTERNO E DEPENDÊNCIA EXTERNO
“*Não há financiamento interno/fundo doméstico para o combate ao HIV*” (Elias G)
“*Posso dizer que a única contribuição do governo no enfrentamento do HIV foram as estruturas físicas e o pagamento de salários dos profissionais que atuam no cuidado ao HIV. Para ser sincero, não é nem investimento no HIV, pois esses profissionais que atuam na ponta, não atendem exclusivamente as PVHIV, atendem a população geral. Não há nenhum centavo do Orçamento Geral do Estado destinado para o combate ao HIV*” (Mila G)
“*Quando o país depende inteiramente dos parceiros, as coisas tendem a não dar certo porque cada parceiro também tem as suas metas e objetivos individuais, que nem sempre vão convergir com os interesses do país. Por exemplo, o Fundo Global é o principal financiador, porém os seus requisitos postos para financiamento nem sempre coincidem com as prioridades traçadas no Plano Estratégico. O que impossibilita o uso desses recursos em algumas atividades do Plano Estratégico Nacional para HIV documento basilar para a resposta ao HIV no país. A tamanha dependência mina a capacidade do país de negociar a forma de uso dos recursos do Fundo Global.* (...) *O governo financia no domínio da saúde, apenas de 6% a 7% a nível nacional*” (Zoe I)
“*A relação do governo com as principais IGSs é de submissão e dependência total por parte do governo. O governo aceita muitas coisas que não deveria aceitar. Por exemplo, alguns parceiros definem, de acordo com os seus critérios, em quais áreas sanitárias do país vão ou não atuar*” (Sofia G)
“*Talvez se não tivéssemos essas ajudas, o governo estaria assumindo a responsabilidade de financiar as ações, mesmo que de forma débil. Em outros países, mesmo com ajuda dessas iniciativas, os governos participam ativamente nas respostas, financiando e coordenando as atividades, mas não é o nosso caso*” (Vitor G)
“*O nosso governo não faz nada. Talvez se pararmos de receber ajuda, o governo vai ter que encarar o problema de HIV no país. Assim eu espero*” (João R)
“*Eu ficaria ressabiada se fosse o governo a financiar, pois não tem comprometimento e há uma instabilidade governativa constante*” (Ravi R)
“*O governo não consegue nem se manter por si só. Tem dificuldade até para pagar os salários dos funcionários, imagina se vai ter condições de arcar com as despesas do combate ao HIV.* (...) *Parece que o governo não tem consciência de que a epidemia do HIV na Guiné-Bissau é problema do Estado guineense, não da comunidade internacional*” (Michel I)
IMPACTO DO FINANCIAMENTO NO PLANEJAMENTO E SUSTENTABILIDADE DAS AÇÕES A LONGO PRAZO
“*São financiamentos a curto prazo, acredito que o planejamento das ações também é de curto prazo e não tem sustentabilidade porque o financiamento não é do governo. Podem desistir a qualquer momento se acharem melhor*” (Ana R)
“*A sustentabilidade das ações está constantemente ameaçada, uma vez que o governo não investe no combate ao HIV, apenas as organizações internacionais, em especial o Fundo Global*” (Tiago R)
“*Não fazem planejamento das ações a longo prazo por causa da instabilidade política e financeira. Se a comunidade internacional decidir se retirar, estaremos largados à própria sorte*” (Pedro R)
“*O Fundo Global, que é o principal financiador, na verdade, o único financiador, já informou que não irá financiar ações de HIV após o ano 2030, por causa da Agenda Global 2030. Aliás, quem garante que não vão mudar de ideia e dizer que o financiamento vai até 2025 por limitações financeiras?*” (Eva R)
“*É comum termos falta de materiais básicos no serviço de urgência do principal hospital do país. O governo fala sempre que está com limitações financeiras, não sei como dará continuidade ao enfrentamento do HIV quando a comunidade internacional deixar de financiar. O financiamento do Fundo Global é por triênio e os planejamentos dos gestores de HIV tem sido feito com base nesses recursos*” (Felipe G)
“*Elaboramos os planos na esperança de que os parceiros irão financiar, mas nunca é uma certeza. Sempre há uma insegurança*” (Carlos G)
“*Não se faz planejamento de longo prazo quando se depende exclusivamente de financiamento externo*” (Elias G)

ARV: antirretrovirais; Fundo Global: Fundo Global de Combate à Aids, Tuberculose e Malária; IGS: iniciativas globais de saúde; PVHIV: pessoas vivendo com HIV; TB: tuberculose.

Os relatos dos participantes convergem na avaliação de que o financiamento internacional, especialmente do Fundo Global, é absolutamente essencial para o combate ao HIV na Guiné-Bissau, respondendo por mais de 90% dos recursos utilizados. Reconheceram também a ausência de um fundo doméstico efetivo, com a contribuição governamental limitada a salários e infraestrutura compartilhada, sem alocação orçamentária específica. Essa dependência quase total de recursos externos é vista como um fator de alto risco, que compromete a continuidade das ações, impõe planejamento de curto prazo e fragiliza a sustentabilidade a longo prazo. Além disso, destacaram que os requisitos dos financiadores nem sempre se alinham às prioridades nacionais, limitando a autonomia do governo e criando uma relação de subordinação às agendas internacionais.

Embora haja consenso sobre a importância do financiamento internacional, os participantes divergem quanto ao papel do governo. Alguns acreditam que, sem ajuda externa, o Estado assumiria maior responsabilidade, enquanto outros demonstram ceticismo quanto à sua capacidade ou compromisso. As opiniões também se dividem sobre a dependência externa: alguns a veem como inevitável, outros criticam a passividade do governo e defendem maior protagonismo. Há ainda diferenças quanto à confiança no governo como potencial financiador, oscilando entre desconfiança e otimismo.

Em relação ao financiamento do combate à epidemia do HIV, a avaliação das contribuições/financiamento das IGSs foi descrita como positiva, principalmente por ser o principal financiador das ações de combate ao HIV na Guiné-Bissau. A mesma realidade também foi verificada em Gana ^21^, onde o Fundo Global responde por substancial parcela do orçamento ao programa de HIV e contribui consideravelmente na melhora da saúde dos ganenses, de modo que possibilitou ao país controlar a epidemia de HIV/aids e progredir no cumprimento das metas dos ODM relacionados à saúde. As ações das IGSs que atuam no enfrentamento de HIV/aids tendem a promover acesso aos serviços de saúde e terapia antirretroviral (TARV), o que resultou na rápida expansão da prevenção, aconselhamento, testagem e tratamento do HIV ^21^ ^,^ ^22^.

O HIV/aids é a quarta principal causa de morte na Guiné-Bissau, porém, recebe uma pequena parcela dos gastos públicos em saúde. O (des)financiamento interno para o combate ao HIV pode ser interpretado como reflexo do baixo investimento interno e alta dependência do financiamento externo para a saúde pública. O país não cumpre o acordo de Abuja (Nigéria), firmado em 2001, pelo qual os chefes de Estado dos países da União Africana assumiram o compromisso de investir pelo menos 15% do Orçamento Geral do Estado (OGE) anualmente. A contribuição do governo nas despesas totais de saúde foi de 7% entre 2018 e 2019 ^23^.

A despesa anual *per capita* com saúde na Guiné-Bissau ainda está abaixo (USD 112) da norma para a Cobertura Universal de Saúde (CUS), indispensável para conquistar o Objetivo do Desenvolvimento Sustentável (ODS) 3 da Agenda 2030 ^23^. Assinala-se que o medo e a preocupação com a insegurança mundial, provocados pela instabilidade econômica, precariedade social e guerras, desviou o foco dos países em relação à saúde. Assim, a meta de atingir mais de um bilhão de pessoas no mundo, amparadas pela CUS, até 2025, se tornou impossível ^24^.

O desfinanciamento interno gerou uma dependência externa por meio da qual um possível corte total dos recursos financeiros externos significaria redução drástica ou inviabilização das ações contra o HIV. Entretanto, a alta dependência externa se traduziu na perda de autonomia das instituições locais. Essa realidade se assemelha a vários países da África Subsaariana, como no caso de Moçambique, país com elevado grau de dependência dos doadores externos, onde o governo é obrigado a seguir as orientações dos doadores no desenvolvimento das políticas públicas nacionais em conformidade com as orientações do *Consenso de Washington*. Tais doadores pressionam e influenciam decisões políticas, como reformas políticas, privatização de empresas públicas e liberalização econômica, limitando, assim, a autonomia para o desenvolvimento e implementação de estratégias domésticas nas políticas nacionais ^25^. Igualmente, no Quênia, foi documentada a perda de autonomia das OSCs para as IGSs em detrimento da continuidade do recebimento dos recursos financeiros ^20^.

A dependência financeira e a perda da autonomia podem minar a capacidade de negociação das instituições locais com as IGSs e promover o desalinhamento com as políticas internas, pois no caso de Malawi, Quênia, Zâmbia e Uganda ^20^ ^,^ ^22^ ^,^ ^26^, as IGSs não consideravam as prioridades de saúde do país em que atuavam, mas sim as suas próprias prioridades.

A fragilidade do governo em financiar os gastos públicos e prover serviços de saúde para sua população desperta a desconfiança em relação aos cuidados mantidos com os recursos governamentais e reforça a concepção da necessidade de ajuda externa. De acordo com a emissora alemã Deutsche Welle ^27^, os “*guineenses não confiam no sistema de saúde pública*” e a “*cooperação internacional é que dita regras ao Estado guineense no setor da saúde pública*”. Dessa forma, a dependência impacta negativamente no planejamento e na sustentabilidade das ações a longo prazo ^25^ e desperta o medo relacionado à possível descontinuidade do financiamento e, consequentemente, à interrupção das ações de enfrentamento ao HIV/aids.

A quarta e última categoria, Utilização dos recursos das IGSs, é formada por duas subcategorias: (i) Aspectos burocráticos e permissibilidade; e (ii) Preferências na canalização dos recursos. Nesta categoria, foram reunidos depoimentos em relação ao aspecto legal e prático do fluxo da canalização dos recursos das IGSs, assim como os motivos de preferências para determinadas formas de canalização dos seus recursos financeiros IGSs, como consta nos relatos do Quadro 5.

**Quadro 5:** Utilização dos recursos das iniciativas globais de saúde (IGS) em Guiné-Bissau.

ASPECTOS BUROCRÁTICOS E PERMISSIBILIDADE
“*A Célula de Gestão envia uma parte dos recursos do Fundo Global para ENDA Santé, ADPP-GB, PLAN Internacional e trabalha em estreita parceria com RENAP.* (...) *Não existe nenhum documento que determine o fluxo de financiamento externo. Tudo depende da capacidade das instituições locais para captar e gerenciar fundos.* (...) *O Plano Estratégico de luta contra HIV* [elaborado pelo SNLS] *é que regula a atuação/de que forma cada IGS pode contribuir. Só podem intervir nas ações definidas no Plano Estratégico*” (Bia G)
“*Qualquer instituição estrangeira que quiser vir atuar no enfrentamento ao HIV, interagindo com a população, precisa ter consentimento do governo, no caso a SNLS e a Direção Geral de Prevenção e Promoção de Saúde. Se for apenas para financiar, podem canalizar seus recursos diretamente para o governo ou para qualquer ONG ou outras organizações da sociedade. Cada ONG tem o seu fluxo burocrático de financiamento. Eles decidem se devem ou não comunicar o governo. Toda intervenção na população precisa estar de acordo com o Plano Estratégico Nacional, por isso que sempre nos comunica* [governo] *e avaliamos se não está fora do que foi definido e, também, para termos conhecimento do que está sendo desenvolvido.* (...) *Recebemos os relatórios dos seus exercícios no país* [IGS]*. No caso dos que fazem intervenções no campo, acho que não costumamos averiguar se o relatório apresentado é verdadeiro*” (Clara G)
“*Depende do tipo de parceria que foi firmado. Se uma ONG firmar uma parceria diretamente com uma entidade internacional, então o recurso é canalizado diretamente para a ONG. Se não for o governo que solicitou o recurso, mas sim a ONG, então tem o direito de recebê-lo diretamente.* (...) *Todas as ONGs que recebem recursos, principalmente recursos internacionais, têm por obrigação legal prestar contas ao governo sobre as suas atividades, pois são fundos de ajuda ao desenvolvimento. Também é uma obrigação social e moral prestar as contas de um recurso doado para o desenvolvimento do país. No nosso caso, todos os projetos de intervenção no domínio da saúde, temos acompanhamento do Ministério da Saúde Pública*” (Dora I)
PREFERÊNCIAS NA CANALIZAÇÃO E OS SEUS MOTIVOS
“*Acho que seria melhor se os financiamentos e as doações fossem diretamente para ONGs e outras organizações da sociedade civil sem passar pelo governo*” (Tiago R)
“*Há instabilidade política e governativa. Quando acontecem problemas no governo, os fundos são bloqueados e tudo para de funcionar, exceto o HIV que fica mais violento. A epidemia de HIV não dá pausa porque o país está com instabilidade política, pelo contrário. Se fosse para atores não governamentais, acredito que, independentemente das instabilidades políticas, as atividades de luta contra o HIV continuariam, pois não dependeria do governo. Se não fosse pela constante instabilidade política que acaba afetando negativamente tudo, eu defenderia que os recursos sejam administrados pelo governo e em colaboração com ONGs que atuam no HIV e TB*” (Paula R)
“*O governo, pela sua estrutura deveria ser o melhor gestor, mas falta muita transparência na administração dos recursos.* (...) *Seria melhor* [as ONGs]*, principalmente às organizações de PVHIV, pois conhecem melhor a realidade. Primeiramente devem ser capacitados para administrar e depois dado autonomia para administrar os recursos*” (Lucas R)
“*Existe muita burocracia que mais atrapalha do que ajuda. Mesmo com dinheiro na conta do governo, os ativistas passam meses sem receber salário.* (...) *Acho que o governo deveria apenas ser o auditor das doações, não o administrador*” (Amílcar R)
“*Sabe como são os nossos governos, má administração e a corrupção são uma das desvantagens e a nossa justiça não funciona. Por isso o Fundo Global disse que tem que ter beneficiários secundários* [ONGs] *que vão gerenciar os fundos*” (Lúcia R)
“*Seria melhor o governo monitorar as atividades das IGSs e tudo que está sendo implementado, mas que não gerencie os recursos. Fazem uso indevido dos recursos. Há muita corrupção. A corrupção não é apenas na área de saúde. É comum ver uma pessoa em cargo público pedindo suborno para aprovação da entrega de organizações internacionais que chegam para investir em alguma área. Em alguns casos, bloqueiam a entrada porque não receberam o suborno. É triste, mas é a nossa realidade*” (Sofia G)
“*Prefiro que os doadores venham e invistam diretamente nas comunidades, sendo eles a gerenciar seus fundos e atividades a serem realizadas. O nosso governo não apoia em nada e não me parece confiável, é corrupto. A RENAP também não é confiável para gerenciar os fundos.* (...) *Prefiro que trabalhem em parceria com RENAP, mas que o pagamento dos funcionários e ativistas sejam diretamente da conta bancária dos doadores para as dos funcionários e ativistas, sem passar pelo governo ou RENAP*” (Alice R)

ADPP-GB: Ajuda de Desenvolvimento de Povo para Povo - Guiné-Bissau; Fundo Global: Fundo Global de Combate à Aids, Tuberculose e Malária; ONG: organização não governamental; PVHIV: pessoas vivendo com HIV; RENAP: Rede Nacional de Associações de Pessoas com HIV; SNLS: Secretariado Nacional de Luta contra SIDA; TB: tuberculose.

Os participantes convergem na percepção de que as intervenções das IGSs devem ser autorizadas pelo governo e alinhadas ao Plano Estratégico Nacional. Destacam a existência de um fluxo formal de aprovação, pelo qual o governo supervisiona oficialmente as ações realizadas e deve receber relatórios das IGSs, assegurando o acompanhamento e a coordenação das atividades. Também reconhecem que as ONGs podem receber recursos diretamente de parceiros internacionais, embora continuem sujeitas à fiscalização governamental. Há consenso quanto à ineficiência, burocracia e falta de transparência do governo na gestão de recursos, com relatos de atrasos, corrupção e ausência de auditoria eficaz. Diante disso, muitos defendem que os recursos sejam preferencialmente canalizados via ONG, consideradas mais próximas da realidade local, mais eficientes e menos suscetíveis a interferências políticas e práticas ilícitas.

Diante do consenso sobre as fragilidades do governo e a preferência pela atuação das ONGs, surgem divergências quanto ao papel ideal do Estado na gestão dos recursos. Alguns participantes defendem que o governo atue apenas como auditor ou supervisor, enquanto outros acreditam que, com reformas e capacitação, ele poderia assumir a gestão principal. As opiniões também variam em relação à confiança nas ONGs: embora a maioria as considere mais eficientes e transparentes, alguns manifestam desconfianças, inclusive em relação às organizações locais, propondo que os doadores gerenciem os recursos diretamente até os beneficiários finais. Além disso, enquanto alguns descrevem um fluxo burocrático formalizado e regulado pelo Plano Estratégico Nacional, outros denunciam práticas recorrentes de corrupção e desvios, que comprometem o controle efetivo do governo.

No que concerne à utilização dos recursos das IGSs, ficou evidente que qualquer instituição não governamental tem permissão para captar recursos financeiros internacionais. No caso do Fundo Global, principal financiador, o recurso fica depositado na conta do governo e depois é canalizado para atores governamentais e não governamentais. Entretanto, fatores como a burocracia excessiva, a falta de transparência e a má administração dos recursos, a corrupção nas instituições governamentais e a instabilidade política e governamental são apontados como motivos das instituições governamentais não serem os principais receptores/administradores dos recursos injetados pelas IGSs, mas sim as ONGs.

A ineficiência e corrupção por parte dos órgãos governamentais de Quênia e Zâmbia, em relação ao gerenciamento dos recursos financeiros das IGSs ^20^, estimulou a condução desses recursos via organismos não governamentais e, consequentemente, proporcionaram maior acesso das partes interessadas a nível comunitário. Nesta ótica, também na África do Sul ^28^, a maioria dos recursos financeiros das IGSs são direcionados pelas ONGs ou Conselho de Pesquisa Médica da África do Sul, sem necessidade de aprovação do Departamento Nacional de Saúde. Assim, comumente, os financiadores externos atuam diretamente com as ONGs. O mesmo fenômeno foi observado na Zâmbia, cujos recursos financeiros das IGSs são encaminhados por diferentes organizações não estatais ^29^.

Pode-se inferir que a defesa dos participantes deste estudo por concentrar os financiamentos das IGSs via ONGs se justifica pelo fato de que a maioria está vinculada direta ou indiretamente às ONGs. Todavia, vale ressaltar que esta forma de financiamento restringe o monitoramento em níveis nacionais ^28^ ^,^ ^29^ e suscita reflexões relevantes sobre até que ponto as IGSs representam as prioridades econômicas e políticas do país em que atuam. Além do mais, retira a soberania do Estado, pois a saúde e a prestação de cuidados constituem parte da função clássica do Estado e dos governos nacionais. Em suma, as IGSs empreendem uma forma de controle na governança global, em que os atores não definem apenas o teor da política, como desenham seus processos de execução e, às vezes, desprezam totalmente o Estado ^29^.

Essa faceta das IGSs pode ser interpretada como filantrocapitalismo, com a transferência da grande parte do poder econômico do Estado para instituições financeiras, como bancos de desenvolvimento e grandes conglomerados e/ou ONGs, o capitalismo encontrou no campo de saúde global o espaço excepcional para progredir sob a rubrica da filantropia. Destarte, nas últimas décadas, a importância dessas instituições multinacionais vem se desenvolvendo rapidamente, com impactos em diversos setores da vida das pessoas e forte interferência nas decisões de saúde pública, que são tomadas em instâncias multilaterais ^30^.

Entende-se o filantrocapitalismo como um instrumento neoliberal que ocorre quando corporações transnacionais disponibilizam financiamentos para projetos filantrópicos, canalizando-os para causas que julgam como prioritárias, de acordo com seus próprios objetivos político-econômicos e interesses particulares. O filantrocapitalismo estimula a privatização de espaços públicos, venalidade da vida humana e hierarquização das pessoas, com vistas a determinar aquelas que são dignas de receber ajuda/cura e as que devem ser deixadas à própria sorte, sustentando as formas de colonialidade por meio de políticas e medidas econômicas fomentadas pelas potências coloniais e seus conglomerados multinacionais, tudo sob a retórica do humanitarismo e da solidariedade ^30^ ^,^ ^31^.

## Conclusão

A partir de distintos atores sociais e políticos, representantes das IGSs, do governo guineense e das OSCs abordados neste estudo, foi possível constatar que a participação das IGSs no enfrentamento da epidemia de HIV/aids em Guiné-Bissau é caracterizada por um paradoxo: de um lado, constata a dependência dos recursos financeiros e técnicos provenientes das IGSs para enfrentamento da epidemia do HIV/aids no país, e do outro, a necessidade de se descolonizar da ajuda externa que, de certa forma, atua como um dispositivo disciplinador.

As IGSs configuraram-se como o principal financiador do combate ao HIV/aids e os recursos fornecidos viabilizaram as ações do governo e das ONGs no planejamento e implementação das ações de controle do HIV/aids no país. Acredita-se que sem o apoio das IGSs, os indicadores de controle da epidemia de HIV (taxa de incidência e morbimortalidade) estariam mais elevados em relação aos indicadores atuais. Fatores como a alta incidência e prevalência do HIV/aids no país, constante instabilidade política e econômica, desfinanciamento interno para combate ao HIV/aids, corrupção e fragilidade das políticas e instituições governamentais, em especial as de combate ao HIV/aids, sustentam a ideia da importância e necessidade da presença das IGSs no combate ao HIV/aids na Guiné-Bissau.

Apesar da necessidade de ajuda externa recursos financeiros das IGSs, a sua dependência e as políticas que lhe envolvem, de modo geral, revelam os tentáculos do neoliberalismo sob a forma do filantrocapitalismo. Nesse sentido, é urgente a necessidade da Guiné-Bissau adotar características de um Estado estável, seguro e com ideais sociopolíticos que possibilitem a compreensão da posição que o país ocupa no contexto geopolítico, bem como a adoção de medidas que instigam sua autonomia e independência da colonialidade (do ser, saber e poder).

As limitações deste estudo estão associadas ao pequeno número dos participantes representantes das IGSs que financiam o enfrentamento do HIV/aids, em comparação com a participação dos membros do governo (gestores do PNLS/HV e/ou coordenadores dos CTAs) e das OSCs. Para mais, encontros únicos e o fato das entrevistas terem ocorrido majoritariamente nos locais de trabalho dos participantes, mesmo sendo em salas privadas, principalmente no caso dos membros do governo, podem ter inibido os (aprofundamentos dos) relatos sobre as limitações e os desafios na gestão/coordenação das políticas e ações nacionais de enfrentamento ao HIV/aids, bem como a relação do governo com essas IGSs. Desse modo, recomenda-se realização de novos estudos com maior participação dos representantes das IGSs que financiam o enfrentamento do HIV/aids, a diversificação das técnicas de coleta de informações e a condução de entrevistas em ambientes que garantam mais discrição e segurança aos participantes.

## Data Availability

Os dados de pesquisa estão disponíveis mediante solicitação à autora de correspondência.
